# Double Versus Single Hepatic Artery Anastomosis in Pediatric Left Liver Grafts: A Reassessment of Outcomes

**DOI:** 10.1111/petr.70212

**Published:** 2025-10-21

**Authors:** Carolina Magalhães Costa, Eduardo Antunes Fonseca, Renata Pereira Sustovich Pugliese, Marcel Ruiz Benavides, Karina Moreira de Oliveira Roda, Rodrigo Vincenzi, Nathália Rangel Porto Travassos, Debora Puzzi Fernandes, Gilda Porta, Irene Kazue Miura, Adriana Porta, Vera Baggio Danese, Teng Hsiang Wei, Fernanda do Carmo Iwase, Mônica Lúcia Rodrigues, Aline Falleiro Freitas, Eliene Novais Oliveira, Cristian Barbieri Victoria Borges, João Seda Neto

**Affiliations:** ^1^ Hepatology and Liver Transplantation Hospital Sírio‐Libanês and Hospital Samaritano São Paulo São Paulo Brazil

**Keywords:** children, hepatic artery thrombosis, liver transplantation, living donation, outcomes

## Abstract

**Background:**

The primary goal of hepatic artery reconstruction is to restore blood flow to the liver graft and its biliary system. Surgical approaches vary in the number of arterial anastomoses, magnification techniques, and anticoagulation strategies. This study analyzes the anatomical approaches used for arterial reconstruction, the incidence of HAT, and associated risk factors.

**Method:**

A retrospective study of 489 primary pediatric LDLT performed between January 2017 and July 2024.

**Results:**

The incidence rates of HAT, early portal vein thrombosis (EPVT), late‐PVT, biliary leak (BL), and biliary stricture (BS) were 1% (*n* = 5), 1.4% (*n* = 7), 4.3% (*n* = 21), 15% (*n* = 73), and 11.9% (*n* = 58), respectively. Double HA anastomosis was performed in 29.4% (119/405) of cases, with LHA‐LHA + MHA‐RHA being the most frequent combination. HAT occurred in four patients (1.4%) with single HA anastomosis and one patient (0.8%) in the double HA group (*p* = 1.00). The rates of BS and BL in the single HA group were 14.7% (*n* = 51) and 12.1% (*n* = 42), respectively, compared to 16% (*n* = 21) and 10.7% (*n* = 14) in the double HA group (BS: *p* = 0.72, BL: *p* = 0.66). Increasing age, CIT, and secondary abdominal closure were associated with higher HAT risk. The overall survival rate was 94.6%, with a median follow‐up of 50.7 months (IQR: 21.1–73).

**Conclusion:**

This study showed a low HAT rate (1%) with increasing age, CIT, and secondary abdominal closure as risk factors. Double HA anastomosis did not reduce biliary complications. The most common HA graft‐recipient combinations in this study were LHA‐RHA for single HA reconstruction and LHA‐LHA + MHA‐RHA for double HA reconstruction.

## Graphical abstract

Our study of 489 pediatric living donor liver transplants demonstrated a low hepatic artery thrombosis rate of 1%. Age and cold ischemia time emerged as significant risk factors. Notably, performing a double hepatic artery anastomosis was safe but did not decrease the incidence of biliary complications such as strictures or leaks.

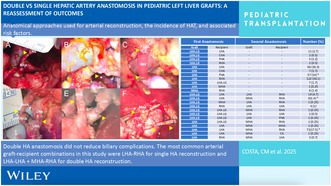

**Abbreviations**
BA: biliary atresia
BD: bile duct
BW: body weight
CA: cystic artery
CHA: common hepatic artery
CIT: cold ischemia time
E‐PVT: early portal vein thrombosis
GRWR: graft‐to‐recipient weight ratio
HA: hepatic artery
HAT: hepatic artery thrombosis
ICU: intensive care unit
LDLT: living donor liver transplantation
LHA: left hepatic artery
LHA.LG: left hepatic artery from the left gastric artery
LL: left lobe
LLS: left lateral segment
L‐PVT: late portal vein thrombosis
MELD: model for end‐stage liver disease
MHA: middle hepatic artery
PELD: pediatric end‐stage liver disease
PHA: proper hepatic artery
PLDLT: pediatric living donor liver transplantation
PRBC: packed red blood cell
RDBW: recipient‐to‐donor body weight ratio
ReTx: retransplantation
WIT: warm ischemia

## 1 Introduction

There are various technical approaches to hepatic artery (HA) reconstruction in pediatric living donor liver transplantation (PLDLT). These approaches differ in the number of arterial anastomoses performed [1], the technique used—such as loupe magnification or operating microscope‐assisted anastomosis [2, 3]—and the choice of anticoagulation strategy. The primary goal of HA reconstruction, regardless of technique, is to restore blood flow to the liver graft and its biliary system. Hepatic artery thrombosis (HAT) can cause severe graft and biliary injury, often requiring further interventions, including retransplantation. Ongoing refinement of surgical techniques is essential to improve outcomes and reduce complications.

A trend analysis based on our group's 20‐year experience with 656 PLDLT [4] revealed a significant decline in the incidence of HAT over time. This reduction coincided with an increased use of double HA anastomosis during graft implantation. In the first 400 patients, the HAT rate was 4.7%, while double HA anastomosis was performed in 8.25% of cases. In contrast, among the last 256 patients, the HAT rate dropped to 0.8%, with double‐artery anastomosis utilized in 19.4% of cases. Eight years have passed, and this report aims to provide a detailed analysis of the anatomical approaches used for arterial reconstruction in our most recent series of PLDLT. Additionally, it seeks to define the incidence of HAT and identify its associated risk factors.

## 2 Methods

A total of 489 primary LDLTs with left liver grafts were performed in patients under 18 years of age at Hospital Sírio‐Libanês, in São Paulo, Brazil, from Jan 2017 to Jul 2024. Data on transplant recipients were collected through a retrospective examination of medical records and a prospectively collected database. The hospital's ethics committee approved this study with protocol number HSL2022‐91.

All procedures were conducted with ABO blood group compatibility. Pediatric patients with end‐stage liver disease requiring LT were initially placed on the waiting list for deceased donor liver transplantation under the Modified PELD system [5]. Assessments of the LT recipients included the investigation of liver disease etiology and severity, imaging studies when applicable, and dental, psychological, and nutritional evaluations.

The donors' pre‐operative evaluations and surgical techniques were reported in previous publications [6] and followed the principles described by Yamaoka et al. [7]. The age limit and highest body mass index accepted for donation were 50 years and 28 kg/m^2^, respectively. Doppler liver ultrasound and abdominal CT‐scan were performed to evaluate vascular anatomy, liver echogenicity, and liver volumetry for left liver grafts (left lateral segment—LLS, and left lobes—LL). Cholangio‐MRI was used to assess the biliary anatomy in cases of previous donor cholecystectomy.

### 2.1 Donor Surgery

A transparenchymal approach, respecting the anatomical boundaries of both the left lateral segment (LLS) and left lobe (LL), was utilized in this series. Parenchymal transection was performed using a Cavitron Ultrasound Surgical Aspirator (CUSA; Cavitron, Stanford, CA) and bipolar electrocautery along predefined anatomical lines, after dissection and isolation of the HA and portal vein (PV) [6, 8].

For LLS resection, the transection line began at the right aspect of the left HA, approximately 1 cm to the right of the falciform ligament, and extended toward the middle of the quadrate lobe, aiming for the left bile duct (BD). For LL resection, the transection followed Cantlie's line (the right aspect of the left hepatic vein), directed toward the middle of the gallbladder fossa and then to the center of the quadrate lobe.

With these anatomical landmarks, the middle hepatic artery (MHA—supplying segment IV), left hepatic artery (LHA), and LHA from the left gastric artery (LHA.LG) were consistently preserved with the retrieved grafts (LL or LLS). Importantly, the transection line never involved the umbilical fissure, thereby minimizing the risk of ischemia to the biliary plate and reducing the likelihood of multiple bile ducts in the graft.

This standardized technique, employed by our group since our first living donor procedure in 1995, prioritizes retrieval of a single BD. BD transection was always guided by two intraoperative cholangiograms: one at the beginning of the procedure to guide the transection plane, and another immediately before dividing the left BD.

During LLS transection, the portal triads to segment IV were ligated within the parenchyma. When necessary to achieve a graft‐to‐recipient weight ratio (GRWR) of less than 4%, additional non‐anatomical reductions—resulting in a hyper‐reduced left lateral segment (HR‐LLS)—were performed during donor surgery. In cases where the left liver graft was supplied by multiple independent arteries, the smallest‐caliber artery was divided first to assess for pulsatile backflow. This assessment guided the decision on whether a single or multiple arterial anastomoses would be required in the recipient.

### 2.2 Recipient Surgery

The liver grafts utilized were the LL, LLS, HR‐LLS. The grafts were implanted using a “piggyback technique”, and the graft's hepatic vein was anastomosed to the recipient's vena cava/hepatic vein as previously described [9]. The graft's PV was anastomosed in an end‐to‐end fashion, either to the recipient's PV trunk or using an interposition vascular graft [10]. The HA was always reconstructed using microvascular techniques with 9–0 or 10–0 nylon sutures (Ethicon, Edinburgh, UK). The OPMI PENTERO 900 and OPMI Vario S88 from ZEISS (Jena, Germany) were the surgical microscopes used in case series. Twenty to 30 min were required to perform an arterial anastomosis, made under 10 times magnification.

A variable number of arteries, from 1 to 3 arterial stumps, supplied the left liver grafts. The arteries supplying the left liver grafts were LHA, segment IV artery or MHA, LHA.LG. The dissection on the recipient side was done high in the liver hilum to achieve as many arterial options as possible for reconstruction. On the recipient side, the arteries used during reconstruction were LHA, MHA, LHA.LG, common hepatic artery (CHA), proper hepatic artery (PHA), and the cystic artery (CA). One or two microsurgical anastomosis were performed during the surgery.

The decision to proceed with double HA anastomosis was based on direct intraoperative surgical assessment rather than on pressure measurements or Doppler studies. Specifically:

- Double arterial anastomosis was only pursued when there was no satisfactory backflow observed after completing the first anastomosis, particularly in grafts with two arteries (notably LL grafts).
- Priority was always given to reconstructing the artery with the larger caliber and best compatibility with the recipient's hepatic artery.
- A second anastomosis was considered only if it could be performed without compromising the integrity or patency of the primary, larger vessel anastomosis.
- No pressure measurements or intraoperative Doppler assessments were used to guide this decision; the approach relied on the surgeon's direct appraisal of arterial backflow and technical feasibility.

There was no intraoperative HAT thrombosis in the studied cohort. Biliary anastomosis was performed either as a duct‐to‐duct or Roux‐in‐Y bilio‐enteric reconstruction.

### 2.3 Post‐Operative Image Studies, Anticoagulation and Management

Doppler US scans were routinely performed on post‐operative day (POD) one to evaluate vascular patency. Further Doppler US scans were performed when clinically indicated (e.g., elevation of liver enzymes, coagulopathy, and fever). Patients who presented altered HA signal findings on Doppler ultrasound underwent upper‐abdominal angio‐computed tomography for comprehensive assessment.

Post‐operative anticoagulation based on an intravenous infusion of heparin was not used and all patients with platelet counts > 50 000/mm^3^ were maintained on acetylsalicylic acid (5 mg/kg/day) for 3 months after the transplant.

Tacrolimus (FK 506, Prograf) and steroids were used for immunosuppression. Details on post‐operative clinical management were previously described [11, 12]. Portal vein thrombosis (PVT) was considered early (e‐PVT) when it occurred within 30 days of the transplant and late (l‐PVT) when it was established after this period. The same time spans were considered for early and late HAT (e‐HAT < 30 days, l‐HAT ≥ 30 days).

### 2.4 Variables Studied

The following variables indicative of the pre‐transplant clinical status and those related to donor and technical aspects during the surgery were investigated:

1. Recipient Numerical Variables on the day of transplant—age (months), weight (kg), BMI, Z‐score for weight‐for‐age, Z‐score for height‐for‐age, bilirubin level, albumin level, creatinine level, International Normalized Ratio (INR), PELD score, and MELD score.
2. Recipient Categorical Variables: indication for transplant, history of prior abdominal surgery, presence of ascites, ICU admission at the time of transplant, dialysis or continuous venovenous hemodialysis (CVVH) before transplant, ventilation requirement before transplant.
3. Donor and Technical Numerical Variables: donor BMI, duration of cold ischemia time (min), warm ischemia time for the recipient (minutes), number of bile ducts in the graft, Number of biliary anastomoses, PRBC transfusion volume (mL/kg)
4. Donor and Technical Categorical Variables: categorized recipient‐to‐donor body Weight Ratio (RDBW), type of graft, graft‐to‐recipient Weight Ratio (GRWR), number of arterial anastomoses, abdominal closure at the end of transplant, type of bile duct reconstruction.

### 2.5 Statistical Analysis

Means and medians were calculated to summarize continuous variables. Parametric comparisons were performed using *t*‐tests, while non‐parametric analyses employed the Mann–Whitney test and Spearman correlation, as data normality could not be confirmed.

Categorical variables are reported as counts and percentages, with group differences assessed using chi‐square or Fisher's exact tests, as appropriate.

Risk factors for HAT were evaluated using univariate logistic regression, with HAT as the dependent variable and various independent variables. Additionally, Spearman's rank correlation was applied for further analysis.

All statistical analyses were performed using R (version 4.2.3), with statistical significance set at *p ≤* 0.05.

## 3 Results

### 3.1 Patients' Demographics and Technical Aspects

During the study period, 489 primary pediatric living donor liver transplants (PLDLTs) were performed, and complete data was available in 477 cases. Of these, 268 recipients (54.81%) were female. The primary indications for liver transplantation (LT) were biliary atresia in 320 cases (65.44%), metabolic diseases in 66 cases (13.50%), and other diseases in 103 cases (21.2%).

The median (IQR) recipient age (months), body weight (kg), and height‐for‐age Z‐score were 12.7 (8.8–29.6), 8.0 (6.7–11.7), and −1.6 (−2.8 to −0.4), respectively. The median (IQR) PELD and MELD scores were 14.0 (6.0–22.0) and 14.0 (7.0–20.0), respectively.

A total of 323 recipients (66.05%) had undergone previous abdominal surgery, including Kasai portoenterostomy before LT. Additionally, 278 patients (56.85%) presented with ascites, 52 (11.04%) were in the ICU, 18 (3.81%) required renal replacement therapy, and 18 (3.82%) were on mechanical ventilation prior to LT.

The donors had a mean (SD) age of 30.6 (7.4) years, body weight of 67.6 (10.6) kg, and BMI of 24.2 (2.8). Table 1 presents the main technical findings observed during the transplantation procedures.

**TABLE 1:** Descriptive analysis of the technical findings observed during the transplantation procedures.

Variable	Findings
RDBW	
Median (IQR)	0.1 (0.10–0.2)
RDBW (receptor/donor) categorized	
≥ 0.1	364 (75.2%)
< 0.1	120 (24.8%)
GRWR (%)	
Median (IQR)	3.12 (2.2–3.9)
GRWR *n* (%)	
≤ 1%	8 (1.6%)
> 1% to 3.9%	365 (74.6%)
≥ 4%	116 (23.7%)
Graft weight (g)	
Mean (SD)	278.4 (62.8)
CIT (min)	
Median (IQR)	36.5 (24–58)
WIT (min)	
Median (IQR)	22 (18–25)
Number of arteries in the liver graft	
Median (IQR)	1 (1–2)
Number of arterial anastomosis	
Mediana (IQR)	1 (1–2)
Number of bile ducts	
Median (IQR)	1 (1–2)
PRBCT (mL/kg)	
Median (IQR)	17.3 (10.2–30.4)
Type of liver graft	
LLS	419 (85.7%)
HR‐LLS	27 (5.5%)
LL	43 (8.8%)
PV reconstruction	
End‐to end	341 (70%)
Vascular graft interposition	146 (30%)
Biliary reconstruction	
Duct‐to‐duct	26 (5.3%)
Hepaticojejunostomy	461 (94.7%)
Primary abdominal closure	465 (97.3%)
Reoperation	115 (23.6%)

Abbreviations: CIT, cold ischemia time; GRWR, graft‐to‐recipient weight ratio; HR‐LLS, hiper‐reduced left lateral segment; LL, left lobe; LLS, left lateral segment; PRBCT, packed red blood cell transfusion; PV, portal vein; RDBW, recipient‐to‐donor body weight ratio; WIT, warm ischemia time.

### 3.2 Anatomical Details and HA Reconstruction

**FIGURE 1:**
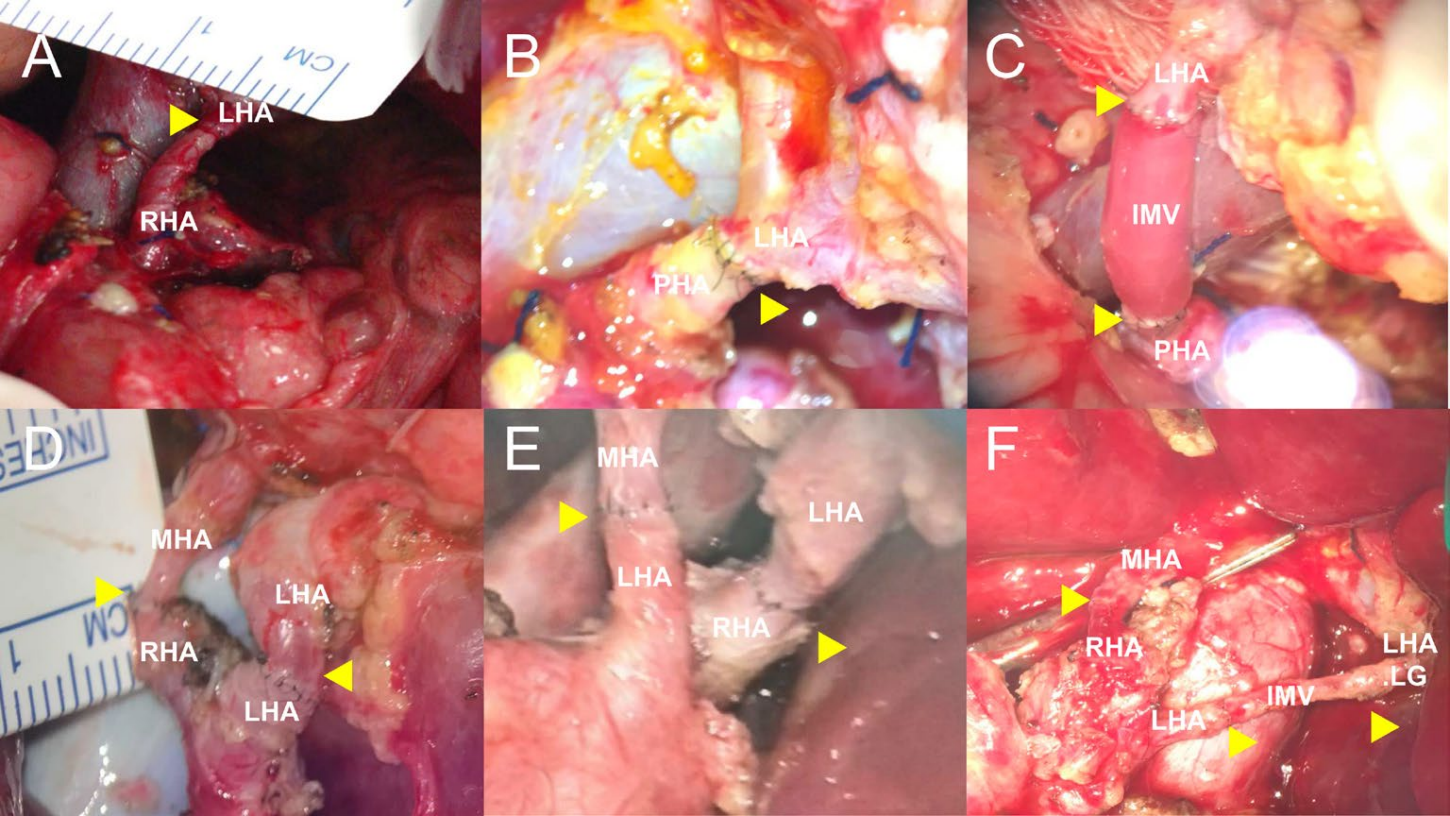
Six examples of microscopic views illustrating different possibilities for donor graft‐recipient arterial reconstruction. Arrowheads indicate the anastomotic plane. (A) LHA‐RHA anastomosis. (B) LHA‐PHA anastomosis. (C) IMV interposition from the LHA to the PHA. (D) MHA‐RHA + LHA‐LHA anastomosis. (E) MHA‐LHA + LHA‐RHA anastomosis. (F) MHA‐RHA + IMV interposition from the LHA‐LG to the LHA. IMV, inferior mesenteric vein; LHA, left hepatic artery; LHA.LG, LHA from the left gastric artery; MHA, middle hepatic artery; PHA, proper hepatic artery; RHA, right hepatic artery.

Among the liver grafts analyzed, 262 had a single artery supplying the left liver graft, 169 had two arteries, and 15 patients had three arteries. In the majority of cases, a single HA anastomosis was performed; 405 cases presented complete anatomical data. The most common graft‐recipient arterial connection was LHA‐RHA, observed in 42.6% (122/286) of cases when a single anastomosis was performed, followed by LHA‐LHA in 23.1% (66/286). Double hepatic artery anastomosis was performed in 29.4% (119/405) of cases, with LHA‐LHA + MHA‐RHA being the most frequent configuration for double anastomoses, used in 59.7% (71/119) of these cases. The anatomical details are presented in Table 2. One observation noted was that in the single anastomosis group, 24.8% (71/286) of the anastomoses were performed to the PHA and CHA. Three patients required interposition of the recipient's inferior mesenteric vein for HA reconstruction (Table 2, Figure 1).

**TABLE 2:** Anatomical details of graft‐recipient single (*n* = 286) or double (*n* = 119) hepatic artery anastomoses in pediatric living donor liver transplantation (405 LT).

First anastomosis		Second anastomosis		Number (%)
Graft	Recipient	Graft	Recipient	
LHA.LG	LHA	—	—	11 (2.7)
LHA.LG	CHA	—	—	2 (0.5)
LHA.LG	PHA	—	—	5 (1.2)
LHA.LG	RHA	—	—	2 (0.5)
LHA	LHA	—	—	66 (16.3)
LHA	CHA	—	—	7 (1.7)
**LHA**	**PHA**	—	—	57 (14)^a^
LHA	RHA	—	—	122 (30.1)
LHA	LHA.LG	—	—	7 (1.7)
LHA	MHA	—	—	1 (0.25)
MHA	RHA	—	—	6 (1.4)
LHA.LG	LHA	LHA	RHA	19 (4.7)
**LHA.LG**	**LHA**	MHA	RHA	6 (1.4)^a^
LHA.LG	MHA	LHA	RHA	1 (0.25)
LHA.LG	RHA	LHA	LHA	4 (1)
LHA.LG	LHA.LG	MHA	RHA	1 (0.25)
LHA.LG	LHA.LG	LHA	LHA	2 (0.5)
LHA.LG	LHA.LG	LHA	PHA	1 (0.25)
LHA	LHA.LG	MHA	RHA	1 (0.25)
LHA	LHA	MHA	MHA	1 (0.25)
**LHA**	**LHA**	MHA	RHA	71 (17.5)^a^
LHA	LHA	MHA	CA	1 (0.25)
LHA	RHA	MHA	LHA	3 (0.7)

Abbreviations: CA, cystic artery; CHA, common hepatic artery; LHA, left hepatic artery; LHA.LG, LHA from the left gastric artery; LT, liver transplantation; MHA, middle hepatic artery; PHA, proper hepatic artery; RHA, right hepatic artery.

^a^ One case in each group required inferior mesenteric vein interposition for arterial reconstruction; the specific site of use is indicated in bold.

Among 71 patients with two or more graft arteries in whom only a single arterial anastomosis was performed, 39.4% of MHA, 13% of left hepatic arteries LHA, and 14% of LHA.LGA were not anastomosed and instead ligated. In the remaining 33% of cases, information regarding the origin of the second, unanastomosed artery was unavailable.

### 3.3 Outcomes and Survival

The median (IQR) ICU and hospital stay for recipients were 10 days (6–18) and 21 days (15–29), respectively. The median (IQR) follow‐up period was 50.7 months (21.1–73). The incidence rates of HAT, EPVT, LPVT, biliary leak (BL), and biliary stricture (BS) were 1% (*n* = 5), 1.4% (*n* = 7), 4.3% (*n* = 21), 15% (*n* = 73), and 11.9% (*n* = 58), respectively. The reoperation rate following LT was 23.5% (*n* = 115), with the most common indications being post‐operative intra‐abdominal bleeding (28.7%), intestinal perforation (20%), bile leak (17.3%), and intestinal obstruction (6%). Overall survival was 94.6%, and the retransplantation rate was 1.6% (*n* = 8). The leading cause of death was infectious complications, accounting for 17 cases (65.3%).

Four patients had early HAT: two underwent retransplantation (one died 27 days after ReTx, the other 14 days post‐ReTx), one underwent early surgical exploration with thrombectomy and continuous heparin infusion, later requiring biliodigestive reconstruction, and one had no intervention and died from severe complications 2 months post‐transplant. One patient developed late HAT, diagnosed 2 years post‐transplant with collateral revascularization, and remained stable without intervention.

### 3.4 Variables Associated With HAT

Table 3 shows the logistic regression performed to evaluate the perioperative risk factors involved with increased risk for HAT. It was identified, with 95% confidence, that each additional month in the recipient's age corresponds to a 2.07% increase in the likelihood of hepatic artery thrombosis. Similarly, each additional minute of CIT corresponds to a 2.9% increase in the likelihood of hepatic artery thrombosis. The secondary abdominal closure also showed a *p*‐value of 0.05. The other studied variables did not determine increased risk of HAT.

**TABLE 3:** Logistic regression of the dependent variable “HAT—Hepatic Artery Thrombosis” in relation to other independent variables.

Variables	Categories	Reference	Hazard ratio	CI (95%)	*p*
Age@LT (months)		—	1.02	1.01; 1.04	**0.004**
BW@LT (g)		—	1.00	1.00; 1.00	0.13
Z‐score recipient (w/a)		—	1.20	0.59; 2.42	0.61
Z‐score recipient (h/a)		—	0.95	0.78; 1.16	0.62
Diagnosis@LT	Metab	BA	4.97	0.69; 35.92	0.11
	Other	BA	1.73	0.15; 19.27	0.66
Previous surgery	Yes	No	0.48	0.05; 4.36	0.52
PELD score		—	0.92	0.84; 1.01	0.09
MELD score		—	0.98	0.78; 1.24	0.89
ICU bound	Yes	No	2.03	0.22; 18.55	0.52
Dialysis	Yes	No	6.62	0.70; 62.42	0.09
Mechanical ventilation	Yes	No	6.60	0.70; 62.28	0.09
Ascites	Yes	No	0.18	0.02; 1.68	0.13
RDBW (receptor/donor) categorized	< 0.1	≥ 0.1	2.04	0.34; 12.35	0.43
Donor BMI (kg/m^2^)		—	1.15	0.84; 12.35	0.38
Type of LL graft	HR‐LLS	LLS			
	LL	LLS	2.47	0.27; 22.61	0.42
GRWR	≤ 1%	≥ 4%			
GRWR	> 1% to 3.9%	≥ 4%	0.47	0.08; 2.86	0.41
CIT (min)		—	1.03	1.01; 1.05	**0.002**
WIT (min)		—	1.03	0.91; 1.16	0.61
Secondary abdominal closure	Yes	No	0.10	0.01; 1.02	**0.05**
PRBCT (mL/kg)		—	1.01	0.98; 1.02	0.52
Number of arterial anastomosis	Double	Single	0.66	0.07; 5.94	0.70

*Note:* Bold values indicate statistical significance (*p* < 0.05).

Abbreviations: BMI, body mass index; BW, body weight; CIT, cold ischemia time; GRWR, graft‐to‐recipient weight ratio; h/a, height‐to‐age; HR‐LLS, hyper‐reduced left lateral segment; ICU, intensive care unit; LL, left lobe; LLS, left lateral segment; LT, liver transplantation; MELD, model for end‐stage liver disease; PELD, pediatric end‐stage liver disease; PRBCT, packed red blood cell transfusion; RDWD, recipient‐to‐donor BW ratio; w/a, weight‐to‐age; WIT, warm ischemia time.

### 3.5 Other Associations

HAT occurred in four patients (1.4%) with a single HA anastomosis and in one patient (0.8%) in the double HA group (*p* = 1.00). Statistical analysis revealed no significant difference in the incidence of biliary complications among the different graft types. Biliary strictures occurred in 40/262 (15.3%) of patients with one artery and one anastomosis, 8/71 (11.3%) of those with two or more arteries and a single anastomosis, and 21/130 (16.2%) of those with two or more arteries and two anastomoses (*p* = 0.63). Similarly, biliary leaks were observed in 36/262 (13.7%), 5/71 (7.0%), and 14/130 (10.8%), respectively, with no statistically significant difference (*p* = 0.27). Spearman's rank correlation was used to assess the relationship between the number of arterial anastomoses and the number of biliary anastomoses in the liver graft (*ρ* = 0.087, *p* = 0.056). The positive correlation (*ρ* = 0.087) suggests that a higher number of arterial anastomoses is associated with a higher number of biliary anastomoses.

## 4 Discussion

The surgical and medical strategies aimed at optimizing HA anastomosis and reducing HAT rates within a transplant team are highly specific. They evolve from past failures and successes, shaping new techniques and interventions. The pursuit of better outcomes is ongoing, and this manuscript is a follow‐up investigation based on three previous studies from our team: (1) HAT was identified as the primary risk factor for death and retransplantation in a cohort of 430 primary PLDLT cases [11]; (2) in a study of 204 LLS donors, up to 40% exhibited double or triple HA inflow anatomy [6]; and (3) a trend analysis demonstrated a reduction in HAT rates over time with the use of double HA anastomoses [4].

In 1996, Ikegami et al. [1] published a landmark study on arterial reconstruction in LDLT. Based on a cohort of 30 LDLT cases, they concluded that although multiple hepatic arteries may supply the liver graft, reconstructing only the largest one—the left hepatic artery—may be sufficient, provided that adequate arterial inflow is achieved after reconstruction, as measured by Doppler ultrasound, and that pulsatile blood flow is observed from the non‐anastomosed stumps. This procedure was widely recognized as safe and has been broadly adopted in LDLT for both right and left liver grafts.

The incidence of multiple arteries is higher in the left lobe grafts, and the arterial diameters of multiple arterial grafts are usually narrower than single arterial grafts [13]. The use of microsurgical anastomosis, implemented since the beginning of our experience, has allowed for a wide range of arterial configurations, as shown in Table 2. This approach requires performing the arterial dissection as distally as possible during recipient surgery to ensure all available options: RHA, MHA, LHA, and the LHA.LG. Equal care must be taken during donor surgery to preserve the longest possible arterial stumps while avoiding injury to the remaining donor arterial anatomy. In left‐sided grafts, this dissection approach typically yields donor‐recipient arterial stumps that are compatible for PLDLT (Figure 1).

In double HA anastomosis, the most common graft‐recipient combination in the present study was LHA to LHA + MHA to RHA (17.5%), followed by LHA.LG to LHA + LHA to RHA (4.7%). Mehta et al. [14] reported that 10% of their cohort had double arterial inflow, with a predominance of right liver grafts (81.7%). Among these cases, only 10 required double HA anastomosis (2.7% in RL and 12.5% in left liver grafts). Harada et al. [15] identified the LHA as the most frequently used artery in single HA anastomosis for left lobe grafts. In 61 cases, double HA was reconstructed 57 times using the LHA and MHA of the graft, though the study did not specify which recipient arteries were utilized. The most detailed anatomical description of left lobe LT comes from Uchiyama et al. [16]. The authors noted that arterial stumps in left hepatic grafts with two HAs were thinner and shorter than those in grafts with a single HA. Of the 71 grafts with two arteries, 47 were reconstructed. Similar to our findings, a wide range of options was used for arterial reconstruction in adults receiving left lobes, including 39 LHA, 20 MHAs, 17 RHA, 6 anterior branches of the RHA, 4 right gastric arteries, 3 right gastroepiploic arteries, 2 posterior branches of the RHA, 1 cystic artery, 1 LHA.LG, and 1 caudate branch.

Similar post‐transplant outcomes—particularly regarding survival, HAT, and biliary complications—have been reported in PLDLT when comparing single versus double hepatic artery anastomoses [4, 17, 18, 19]. However, a recent study by Harada et al. [15] involving 258 left liver grafts (LL and LLS) identified partial arterial reconstruction as the only significant independent risk factor for postoperative anastomotic biliary stricture in Cox regression analysis. Similarly, other studies have reported comparable findings [14, 16, 20] in cohorts ranging from 31 to 261 LT cases, including a study from our team on 670 primary pediatric LT cases, which showed a trend toward fewer biliary complications with double HA anastomosis [21]. The present study provides further insight into this issue, finding no protective association between double hepatic artery anastomosis and biliary complications (BS or BL) in a PLDLT cohort with a high prevalence of double anastomoses (27.4%, 131/477 cases). Similarly, performing a single anastomosis in liver grafts with two arteries did not result in increased rates of biliary complications.

For events with low frequency, identifying factors associated with increased risk is challenging. Moreover, the low rates of HAT in patients with single (1.4%) or double (0.8%) HA anastomoses suggest that advancements in surgical technique, post‐operative care, and follow‐up have collectively contributed to achieving low HAT rates over time, from 3.2% (21/656 initial cases) [4] to 1% (5/486) in the present report. Age, cold ischemia time (CIT), and secondary abdominal closure were independently associated with HAT in this cohort. Although secondary abdominal closure—often required in cases with high GRWR—has traditionally been associated with increased risk of vascular complications due to size mismatch, delayed closure, or reintervention [22, 23], a recent study of 664 pediatric liver transplants (8.7% with secondary closure) found no significant difference in vascular complication rates compared to primary closure [24]. While longer CIT reflects prolonged preservation, leading to marked ischemia–reperfusion injury and an increased risk of HAT [25], the association between increasing age and HAT risk is less intuitive. However, in our previous HA study [4], receiving a graft with GRWR < 1% was independently associated with HAT risk. Notably, these smaller grafts were predominantly transplanted into older pre‐adolescent and adolescent recipients. Indeed, the arteries of children in this age group are typically more fragile and prone to intimal dissection during hilum preparation, making reconstruction more challenging and often requiring greater technical precision.

The paradigm shift in our practice from single to double HA anastomosis emerged as a strategy to reduce HAT rates, which were associated with poorer patient and graft survival as well as increased perioperative morbidity. Microvascular anastomosis using surgical microscopes had already been standard practice in our team since the first PLDLT. The anatomical recognition that up to 40% of left‐sided grafts had two arterial inflows enabled the routine use of double arterial anastomoses whenever feasible, prioritizing the connection of the larger vessels first—provided the second anastomosis did not compromise the integrity of the primary reconstruction. However, in most cases, vessel calibers remain small, and the present data show that only 24.8% (71/286) of single arterial anastomoses were performed in the CHA or PHA.

Our study demonstrated a low hepatic artery thrombosis (HAT) rate (1%), with increasing age, CIT, and secondary abdominal closure identified as risk factors. Double hepatic artery (HA) anastomosis was performed in 27.4% of cases but was not associated with lower rates of biliary complications (BS or BL). Successful double HA reconstruction requires high hilar dissection on the recipient side and thorough arterial dissection on the donor side. The most common arterial graft‐recipient combinations in this study were LHA‐RHA for single HA reconstruction and LHA‐LHA + MHA‐RHA for double HA reconstruction.

## Conflicts of Interest

The authors declare no conflicts of interest.

## Data Availability

The data that support the findings of this study are available on request from the corresponding author. The data are not publicly available due to privacy or ethical restrictions.
